# *SLC12A2* mutations cause NKCC1 deficiency with encephalopathy and impaired secretory epithelia

**DOI:** 10.1212/NXG.0000000000000478

**Published:** 2020-07-02

**Authors:** Tommy Stödberg, Måns Magnusson, Nicole Lesko, Anna Wredenberg, Daniel Martin Munoz, Henrik Stranneheim, Anna Wedell

**Affiliations:** From the Department of Women's and Children's Health (T.S.), Department of Molecular Medicine and Surgery (M.M., N.L., H.S., A. Wedell), Science for Life Laboratory (M.M., H.S., A. Wedell), Department of Medical Biochemistry and Biophysics (A. Wredenberg), and Department of Clinical Neuroscience (D.M.M.), Karolinska Institutet; and Department of Pediatric Neurology (T.S.), Centre for Inherited Metabolic Diseases (N.L., A. Wredenberg, H.S., A. Wedell), and Department of Neuroradiology (D.M.M.), Karolinska University Hospital, Stockholm, Sweden.

## Abstract

**Objective:**

To describe the phenotype in 2 sisters with a rare constellation of neurologic symptoms and secretory impairments and to identify the etiology by the use of whole-genome sequencing (WGS).

**Methods:**

After an extensive workup failed to reveal the cause of disease, in a girl with a previously not reported phenotype, WGS of the proband, her diseased older sister, an older healthy brother, and their parents was performed, and potentially pathogenic variants were analyzed.

**Results:**

The proband and her older sister both presented with neonatal *Staphylococcus aureus* parotitis, apneas, disappearance of the Moro reflex, and hypotonia. The proband survived. Her brain MRI showed white matter and basal ganglia abnormalities, and CSF damage biomarkers were increased. At age 8 years, she exhibits a constellation of symptoms including severe neurodevelopmental disorder, hearing impairment, gastrointestinal problems, and a striking lack of tear fluid, saliva, and sweat. Her respiratory mucosa is dry with potentially life-threatening mucus plugging. Through WGS, 2 loss-of-function variants in *SLC12A2* were identified that follow an autosomal recessive inheritance pattern.

**Conclusions:**

Taken together with a single previously reported case and the close resemblance to the phenotypes of corresponding mouse models, our study firmly establishes biallelic variants in *SLC12A2* as causing human disease and adds data regarding the neurologic phenotype.

The Na-K-Cl cotransporter sodium-potassium-chloride cotransporter 1 (NKCC1), encoded by the *SLC12A2* gene, is widely expressed in neurons and secretory epithelial cells of multiple organs.^1–3^ *SLC12A2*, located on chromosome 5q23.3, has only recently been implicated in human disease. The SLC12 family of solute carriers consists of 9 electroneutral cation-chloride cotransporters (CCCs) involved in epithelial ion transport, gamma-aminobutyric acid (GABA)-mediated neurotransmission, and cell volume regulation.^4^ Another SLC12 member, the potassium-chloride cotransporter 2 (KCC2), encoded by *SLC12A5*, is the main chloride exporter in neurons.^5^ As a neuronal chloride importer, NKCC1 is in dynamic balance with KCC2, thus regulating the intraneuronal chloride concentration on which GABA neurotransmission and neuronal excitability depends.^2,6^

Four of the CCCs have previously been linked to monogenic disease in humans,^7–10^ of which 2 show a neurologic phenotype.^9,10^ Autosomal recessive early infantile epileptic encephalopathy results from pathogenic variants in *SLC12A5*.^9^ Biallelic variants in *SLC12A6*, encoding KCC3, are linked to agenesis of corpus callosum with peripheral polyneuropathy, also called Andermann syndrome.^10^ For *SLC12A2*, there are mouse models exhibiting phenotypes related to NKCC1 deficiency in neurons and epithelia.^4^ The first human case of a homozygous deletion in *SLC12A2* was recently described.^11^ In this study, we report the detailed characterization of 2 sisters with an NKCC1 deficiency syndrome due to novel biallelic variants in *SLC12A2*.

## Methods

### Subject recruitment

The proband was referred to our hospital at age 9 days. An extensive etiologic workup was performed before her inclusion as a research subject. The proband's 21-month-older sister had been treated and died at another hospital, and fibroblasts were preserved for future diagnostics.

### Standard protocol approvals, registrations, and patient consents

Written informed consent was obtained from the parents including a consent to disclose photographs of the proband. The study was approved by The Regional Ethical Review Board in Stockholm, Sweden.

### Molecular genetic analyses

#### DNA and RNA isolation

See supplementary material (links.lww.com/NXG/A277).

#### Whole-genome sequencing

Whole-genome sequencing (WGS) of the proband, her 2 siblings, and their parents was performed to a sequencing depth of 30× mean coverage using a HiSeq X sequencing instrument and a PCR-free library preparation method. The resulting sequences were analyzed using the Mutation Identification Pipeline (MIP) as previously described.^12^ All called variants were scored and ranked using the MIP weighted sum model, which uses multiple parameters, but emphasizes mendelian inheritance patterns, conserved, rare, and protein-damaging variants. The genome was analyzed for potential pathogenic variants in exons or splice regions.

#### Molecular analysis of *SLC12A2*

See supplementary material (links.lww.com/NXG/A277).

### Data availability

The data that support the findings of this study are available from the corresponding author on reasonable request.

## Results

### Clinical summary

**Figure 1:**
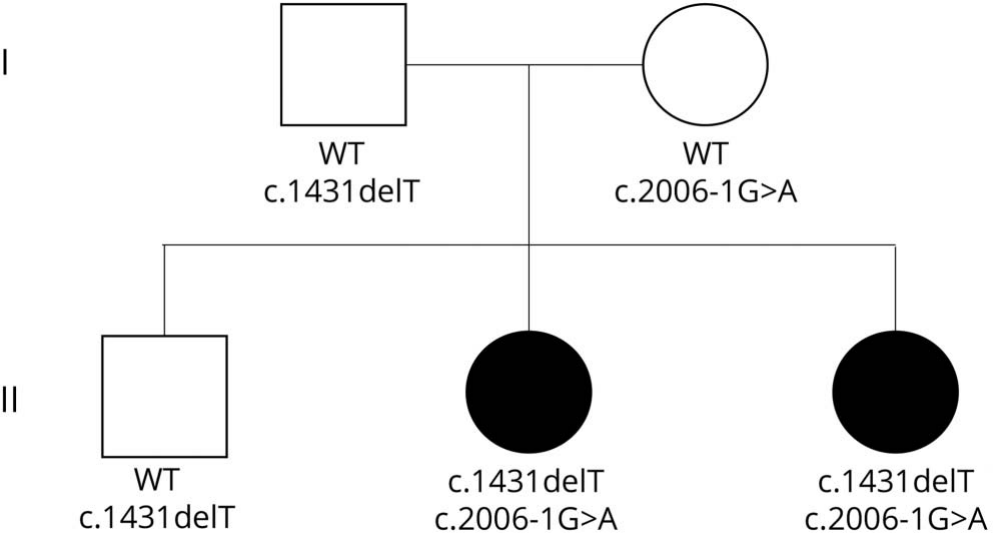
Pedigree of the family with *SLC12A2* variants Variant nomenclature follows reference transcript NM_001046.

The proband (II:3) (figure 1) is an 8-year-old girl and the third child of nonconsanguineous parents of Swedish descent. In addition to the deceased older sister (II:2), she has a healthy older brother (II:1).

The older sister was born 21 months before the proband, full term with birth weight 3.0 kg after a normal pregnancy. She was described as hypotonic. At age 9 days, she presented with bacterial parotitis confirmed by ultrasound and growth of *Staphylococcus aureus* in pus from the parotid gland. She was tired but afebrile, infection parameters were only slightly elevated, and blood cultures negative. The infection was well controlled by antibiotics, but the patient remained hospitalized due to apneas, muscle hypotonia, and feeding problems. It was noted that the Moro reflex that was normal during the first week of life later could not be elicited. During the third week, apneas of unknown cause worsened. A chest x-ray was normal. No viral or infectious agents were detected in CSF, blood, urine, feces, or nasopharynx. Infectious parameters were normal, and CSF showed no pleocytosis and normal albumin. Brain imaging was not performed. At age 22 days, the patient died of asystole during assisted ventilation. Fibroblasts were preserved. Autopsy revealed aspiration of ventricular content into the lungs. There were no malrotation or other internal malformations.

**Figure 2:**
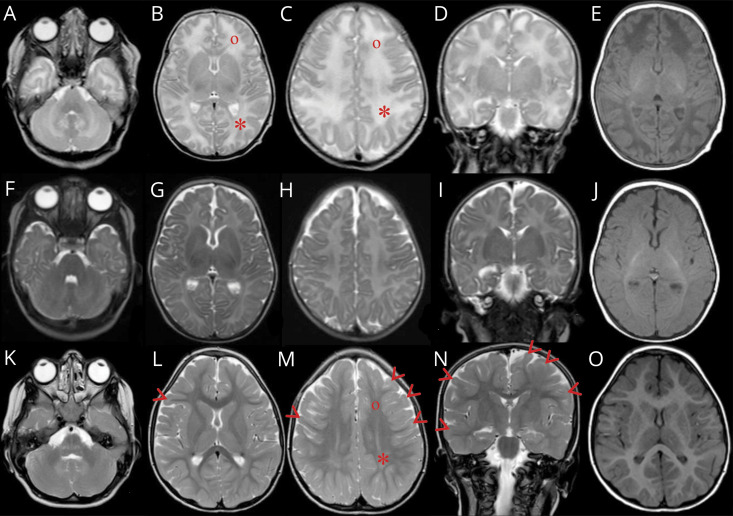
MRI of the index patient (II:3) T2-weighted MRI at ages 23 days (A–D), 2 months (F–I), and 4 years (K–N) and T1 at corresponding age (E, J, and O). Peripheral white matter in the parietal (*) compared with frontal (°) lobes shows increased signal at 23 days (B and C) that persists in parietal lobes at 4 years (M). At age 4 years, subcortical frontal and temporal white matter (arrow heads) (L–N) shows persistant high signal for age when it should have resolved by age 3 years.

**Figure 3:**
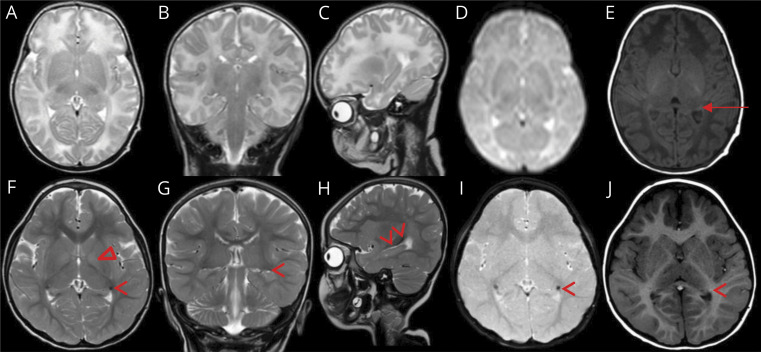
MRI of the index patient (II:3) Images at ages 23 days (A–E) and 4 years (F–J). T2-weighted MRI in axial (A and F), coronal (B and G), and sagittal plane (C and H), axial GRE imaging (D and I), and T1-weighted imaging in axial plane (E and J). At age 4 years, a persistent high signal is perceived in the basal part of the bilateral globus pallidi (Δ) (F), which is not seen at age 23 days (A). The caudate part of the caudate nuclei (arrow head) presents low signal on T2 in all 3 planes (F–H) including GRE imaging (I) with high signal on T1 (J) compared with normal appearance at age 23 days (A–E). Only the normal mature lateral geniculate body of the thalamus (arrow) is seen at age 23 days just medially to the caudate part of the caudate nucleus (E). GRE = gradient echo sequences.

The proband was born full term with birth weight 3.2 kg after a normal pregnancy. Delivery was unremarkable. She was transferred to a neonatal unit due to hypotonia and difficulties breastfeeding. The following course was characterized by bacterial parotitis and *S aureus* septicemia presenting at day 5, central and obstructive apneas, disappearance of the Moro reflex, and muscle hypotonia. The similarity with the symptoms of the deceased older sister was striking. The patient was admitted to our hospital's pediatric intensive care unit on day 9. She was treated with antibiotics, continuous positive airway pressure, oxygen, caffeine citrate, and frequent inhalations of sodium chloride. The infection was promptly controlled, but apneas persisted until she could be discharged to her local hospital at age 7 weeks. Because of the history of the older deceased sister, an extensive workup for immunodeficiency and metabolic disease was started. Results came back negative as did a genetic test for cystic fibrosis. A neurologic workup was prompted by the muscle hypotonia, central apneas, and the lack of Moro reflex. MRI (figures 2, A–E and 3, A–E) and magnetic resonance spectroscopy (MRS) (supplementary material, figure e-1, A and B, links.lww.com/NXG/A277) of the brain revealed white matter and basal ganglia abnormalities. Lumbar puncture showed elevated albumin and increased damage biomarkers in CSF (supplementary material, table e-1).

**Figure 4:**
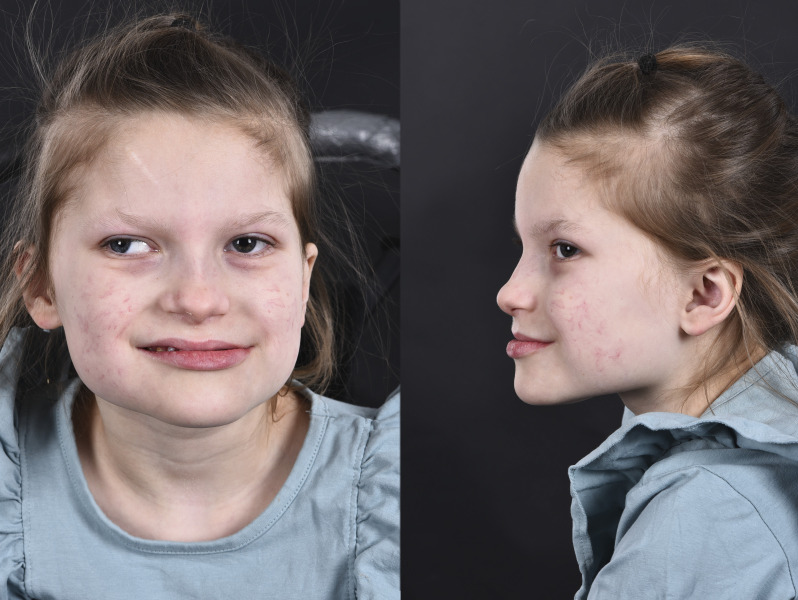
Photographs of the index patient (II:3) Photographs at age 8 years, showing dysmorphic facial features and strabismus. She has a broad and square lower face, broad chin with mandibular prognathia, wide mouth, and narrow forehead.

The continued course has been without severe infections or other dramatic events. The patient shows dysmorphic facial features (figure 4). Her head circumference and length are around −2.5 SD and weight −3.5 SD. She has severe intellectual disability and does not speak or walk. She has a hearing impairment and a striking lack of tear fluid, saliva, and sweat. Her respiratory mucosa is dry, which necessitates frequent inhalations of hypertonic sodium chloride to prevent life-threatening mucus plugs. She has constipation and an intestinal malrotation. Follow-up MRI investigations of the brain have been performed at 2 months (figure 2, F–J) and 4 years (figure 2, K–O and 3, F–J) and CSF analyses at 4 months and 4 years (supplementary material, table e-1, links.lww.com/NXG/A277).

### Molecular genetic analyses

When the proband was 6 years of age, WGS of all family members was performed. The cause of her and her older sister's disease was still unknown, and the parents where planning another pregnancy. Analysis of sequencing data did not reveal any potential pathogenic variants in 870 known metabolic disease genes. When expanding the analysis to the whole genome, no potential disease-causing variants that fit with an autosomal recessive inheritance were detected in any known disease-causing gene. When moving to genes not previously linked to monogenic diseases, 2 highly interesting variants were detected in *SLC12A2*. At the time, no patients with *SLC12A2* mutations had been reported. No other potential disease-causing variants that fit the segregation among the 5 family members were detected. Both variants were ranked high by the MIP-weighted sum model. The index patient and her deceased older sister were compound heterozygotes for these variants. Variant 1, inherited from the mother, alters a canonical splice acceptor site, c.2006-1G>A. The second, paternally inherited variant, is a 1 base deletion, c.1431delT, causing a frameshift in exon 8. Both variants are thus severe loss-of-function mutations that are clearly incompatible with production of a normal NKCC1 protein. Both variants are absent in gnomAD^13^ v2.1.1 as well as in our in-house frequency database consisting of more than 2,600 clinical whole genomes. According to the American College of Medical Genetics guidelines both variants are classified as likely pathogenic. Combined Annotation-Dependent Depletion scores^14^ of 24 and 34, respectively, support the deleterious effects of the variants. The observed/expected gene constraint metric (oe) of 0.19 in gnomAD indicates that *SLC12A2* is highly intolerant to loss-of-function variants. Sanger sequencing confirmed the segregation in the family.

**Figure 5:**
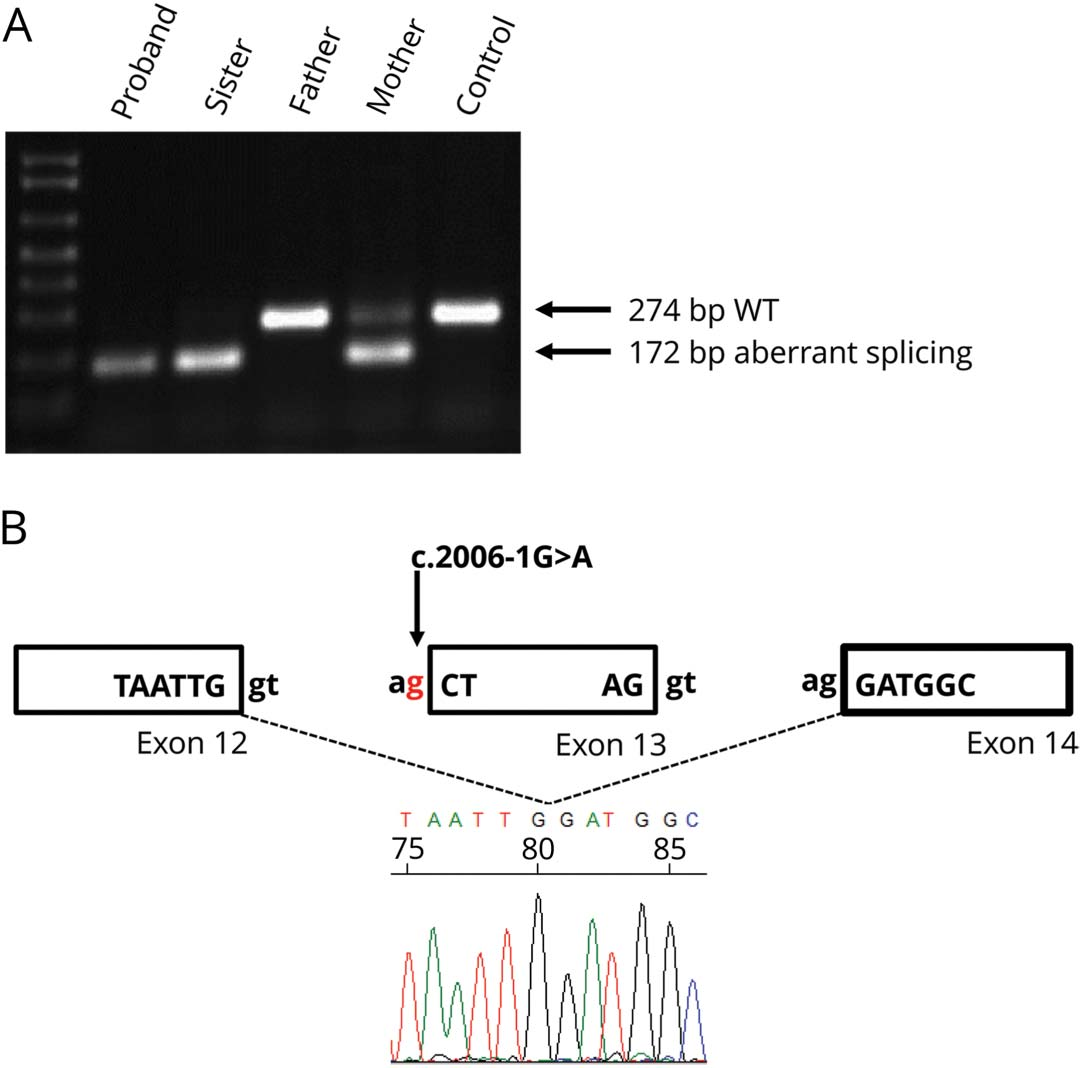
cDNA studies on fibroblast cell lines from patients and parents (A) PCR products run on an agarose gel following amplification of exons 12–14 of SLC12A2. Wild-type products are 274 bp in length and products lacking exon 13 are 172 bp long. (B) Schematic diagram and partial electropherogram showing skipping of exon 13 as a result of a defect in splicing due to the presence of the c.2006-1G>A variant. cDNA = complementary DNA.

As the heterozygous c.2006-1G>A variant is positioned in the splice acceptor site, it is predicted to affect normal splicing of exon 13. Splicing of the *SLC12A2* transcript (NM_001046.3) was examined by PCR amplification of complementary DNA from exon 12 to exon 14. A PCR product of 172 bp was amplified, which was 102 bp shorter than the expected wild-type allele, and following Sanger sequencing, it was subsequently found to entirely lack exon 13 (figure 5). Sequencing of exon 8 from both the patients' and father's complementary DNA showed a lack of expression from the allele carrying the deletion, (c.1431delT) indicating nonsense mediated decay of this transcript (data not shown).

## Discussion

We report 2 sisters exhibiting a complex phenotype caused by NKCC1 deficiency due to novel compound heterozygous variants in *SLC12A2*. Taken together with a recently published case of homozygous deletion in *SLC12A2*, and the close resemblance to the phenotypes of *SLC12A2* knockout mouse models, our study firmly establishes *SLC12A2* as a disease gene in humans and adds new data regarding the neurologic phenotype.

Our proband and her sister's neurologic symptoms as neonates were similar. The proband's CSF findings suggested recent and/or ongoing neuronal damage that in our opinion could not be explained by hypoxia-ischemia or septicemia. She had short apneas and desaturations but was otherwise stable and not clinically septic. The ensuing neurodevelopment of the proband has been severely impaired in both cognitive and motor domains but without regression.

GABA, the main inhibitory neurotransmitter in the mature brain, is excitatory in immature neurons due to high intraneuronal [Cl^−^]. This is caused by the high expression of NKCC1 and the immaturity of KCC2.^2,15^ During nervous system development, excitatory GABA acts as a trophic factor and affects proliferation, migration, differentiation, synapse maturation, and cell death,^6^ thus influencing brain development and CNS network formation. In the CNS of *SLC12A2* knockdown mice, neuronal proliferation is decreased, and dendrite development delayed.^16^ Based on this, the severe neurodevelopmental impairments in our proband could be of prenatal origin. This is supported by the evolution of the early and follow-up MRI findings (figure 2), together suggestive of hypomyelination. Alternatively, the neonatal white matter findings could, at least partially, be due to a more acute neurometabolic encephalopathy with cytotoxic edema. The latter is supported by the very high damage biomarkers in CSF (supplementary material, table e-1, links.lww.com/NXG/A277), and the MRI at 2 months showing resolution of the supratentorial white matter findings and wider ventricles and sulci compared with day 23 (figure 2, F–J), implying edema at day 23. MRS indicates loss of neurons but cannot date this (supplementary material, figure e-1). After the neonatal period, the disease course has not demonstrated a progressive decline. MRI at 4 years (figure 2, L–N) showing increased T2 signal in the subcortical white matter of the frontal and temporal lobes could indicate delayed myelination or cortical developmental anomaly as the signal should have normalized by no later than age 3 years. Furthermore, no decreased signal on T2 and gradient echo sequences, suggestive of hemorrhage or calcifications, was shown at age 23 days (figure 3, A–D) but developed by age 4 years (figure 3, F–I) suggestive of mineral deposition in the caudate of the caudate nucleus as it trajects superior in the wall of the temporal horn of the lateral ventricle both superior and lateral to the fornix of the hippocampus and lateral geniculate body of the thalamus. The CSF findings at 4 months and 4 years suggest some degree of ongoing neurodegeneration and disruption of the blood-brain barrier (supplementary material, table e-1).

In brainstem respiratory nuclei of rats, NKCC1 and KCC2 expression shows developmental decrease and increase, respectively, during early postnatal life.^17^ The resulting rapid increase of GABA inhibition could destabilize the respiratory network during a critical period. We hypothesize that NKCC1 deficiency contributed to the central apneas in our patients through enhanced neonatal respiratory inhibition or impaired prenatal respiratory network formation. Another striking, and otherwise rare, symptom in our patients is the early permanent loss of the Moro (startle) reflex, also with a potential relation to NKCC1 deficiency in involved brainstem nuclei.^18^

NKCC1 is expressed in the basolateral membrane of epithelia in various organs, transporting chloride across the basolateral membrane into the epithelial cells where chloride then can be secreted.^3^ The severely reduced saliva in our proband closely resembles the deficient saliva secretion from the parotid gland shown in *SLC12A2* knockout mice.^19^ The *S aureus* parotitis of the proband and her sister was assumingly caused by obstruction of the parotid duct due to reduced secretion. Adult *SLC12A2* knockout mice do not show the cystic fibrosis–like lung phenotype seen in our proband, presumably due to compensatory mechanisms not found in tracheal epithelia from adult rabbits and humans.^20,21^ The impaired hearing and the gastrointestinal problems of our proband are likewise described in *SLC12A2* knockout mice.^22–24^

Recently, a boy with a homozygous 22-kb deletion in *SLC12A2* due to paternal parental isodisomy was described as the first convincing case of *SLC12A2*-related disease.^11^ A close resemblance with our proband includes reduced sweat, saliva, and tear fluid, cystic fibrosis–like respiratory problems, intestinal malrotation, a severe neurodevelopmental disorder, hearing impairment, and similar dysmorphic facial features. Whether the boy had any neurologic symptoms in the neonatal period is not reported. Because it is not clearly stated, the neonatal course was presumably not as dramatic as in our patients. The only MRI performed at 9 months is described as showing decreased brain volume involving both gray and white matter, whereas MRI at 4 years in our proband did not show atrophy. Whether CSF analysis was performed is not stated. There are thus to date only 3 patients reported, all of whom are homozygous for completely inactivating mutations in *SLC12A2*. Further delineation of genotype-phenotype relationships, including potential consequences of partially inactivating mutations, awaits identification of additional cases.

In 2016, a patient carrying a heterozygous de novo truncating variant in *SLC12A2* was reported.^25^ With first symptoms at 6 months, this female patient developed a multisystem disorder with respiratory, circulatory, gastrointestinal, and endocrine/metabolic dysfunction. Although the patient's disorder has little resemblance with the phenotype of the 3 known patients with homozygous loss of function mutations or *SLC12A2* knockout mice, the possibility remains that the truncating variant was responsible for the phenotype through a different, gain-of-function mechanism.

We report 2 sisters, with biallelic loss-of-function variants in *SLC12A2* causing an NKCC1 deficiency syndrome with a characteristic constellation of neurologic symptoms and secretory impairment in multiple organs. Identification of additional cases is needed to assess phenotypic variability and genotype-phenotype correlations. The relative roles of prenatal and postnatal disease mechanisms, and whether acute or progressive encephalopathy is part of the syndrome, need to be further explored because this can affect treatment possibilities.

## Glossary

CCC: cation-chloride cotransporter
KCC2: potassium-chloride cotransporter 2
MIP: Mutation Identification Pipeline
MRS: magnetic resonance spectroscopy
NKCC1: sodium-potassium-chloride cotransporter 1
WGS: whole-genome sequencing
